# An ultrasonographic evaluation of skin thickness in breast cancer patients after postmastectomy radiation therapy

**DOI:** 10.1186/1748-717X-6-9

**Published:** 2011-01-24

**Authors:** Sharon Wong, Amarjit Kaur, Michael Back, Khai Mun Lee, Shaun Baggarley, Jiade Jay Lu

**Affiliations:** 1 National University of Singapore, Yong Loo Lin School of Medicine, 21 Lower Kent Ridge Road, 119077, Singapore; 2 Nanyang Polytechnic, School of Health Sciences, 180 Ang Mo Kio Avenue 8, 569830, Singapore; 3 Northern Sydney Cancer Centre, Royal North Shore Hospital, St Leonards, New South Wales 2065, Australia; 4 National University Cancer Institute, Department of Radiation Oncology, National University of Singapore, 1E Kent Ridge Road, Tower Block, Level 7, 119 228, Singapore

## Abstract

**Background:**

To determine the usefulness of ultrasonography in the assessment of post radiotherapy skin changes in postmastectomy breast cancer patients.

**Methods:**

Patients treated for postmastectomy radiotherapy in National University Hospital (NUH) and Tan Tock Seng Hospital (TTSH), Singapore between January 2004- December 2005 was recruited retrospectively. Ultrasound scan was performed on these Asian patients who had been treated to a total dose of 46-50 Gy with 1 cm bolus placed on the skin. The ultrasound scans were performed blinded to the RTOG scores, and the skin thickness of the individually marked points on the irradiated chest wall was compared to the corresponding points on the non-irradiated breast.

**Results:**

The mean total skin thickness inclusive of the epidermis and the dermis of the right irradiated chest wall was 0.1712 mm (± 0.03392 mm) compared with the contra-lateral non-irradiated breast which was 0.1845 mm (± 0.04089 mm; p = 0.007). The left irradiated chest wall had a mean skin thickness of 0.1764 mm (± 0.03184 mm) compared with the right non-irradiated breast which was 0.1835 mm (± 0.02584 mm; p = 0.025). These independent t-tests produced a significant difference of reduced skin thickness on the right irradiated chest wall, p = 0.007 (p < 0.05) and left irradiated chest wall p = 0.025 (p < 0.025) in comparison to the non-irradiated skin thickness investigating chronic skin reactions. Patients with grade 2 acute skin toxicity presented with thinner skin as compared to patients with grade 1 (p = 0.006).

**Conclusions:**

This study has shown that there is a statistically significant difference between the skin thicknesses of the irradiated chest wall and the contra-lateral non-irradiated breast and a predisposition to chronic reactions was found in patients with acute RTOG scoring of grade1 and grade 2.

## Introduction

Breast cancer is the most commonly diagnosed cancer and the leading cause of cancer deaths among women worldwide [1]. In addition to the acknowledged advances in surgical and medical therapies, the role of radiotherapy continues to remain important for all stages of breast cancer. While its role as adjuvant therapy in selected patients undergoing mastectomy for stages I and II disease is currently evolving, it has however, become an essential component of the combined modality approach for stage III disease. Postmastectomy radiotherapy (PMRT) to the chest wall and to the regional lymphatics has shown to decrease locoregional recurrence and increase survival for women with large tumors and/or node-positive disease [2-5]. These studies showed that PMRT not only reduced local regional recurrence rates but also improved disease free and overall survival rates in premenopausal patients receiving chemotherapy.

In spite of the advances in radiotherapy techniques, early and late adverse effects after breast irradiation are reported in a range of organs and tissues. Some of these adverse effects include ischemic heart disease, pneumonities and pulmonary fibrosis, erythema, telangiectasia and ulceration of the skin [6,7] with skin being the most commonly affected area during breast cancer irradiation. While early effects can heal almost completely, the severe delayed changes that follow such as dermal atrophy, fibrosis, retraction and susceptibility to necrosis remain, and may affect the function and physical properties of the skin [8,9]. Acute skin complications in postmastectomy patients have been well studied [10-12], but little is known about risks of long term skin complications and cosmesis of these patients. This forms the basis of our study.

Long term skin complication such as fibrosis is a common late side effect of radiotherapy treatment for breast cancer patients and is considered to be a dose-limiting factor during the therapy. Quantitative and objective assessment of late skin reactions is helpful for oncologists and clinicians to estimate the efficacies of radiotherapy regimens or prediction of cosmetics outcome of these patients

Previous studies on radiation induced skin effects have shown that skin assessments have been either descriptive or have used subjective parameters for scaling radiation effects [10,11]. The visual assessments of skin condition are carried out subjectively by the examining physician and it is well known that the estimation of the visible changes by different examiners can be significantly biased [13]. While the European Organisation for Research and Treatment of Cancer (EORTC) and Radiation Therapy Oncology Group (RTOG) [14] have an elaborate scoring system for acute skin reactions, evaluation of late skin changes are more descriptive, and there is no scoring to convey the amount of edema, indurations and fibrosis that may be present [12].

As such, a quantitative assessment and documentation of late postradiation skin reactions are important for following up of PMRT patients.

Quantitative methods that have been used to monitor skin changes following radiation therapy include direct evaluation of the mechanical properties of irradiated skin by the measurement of tensile strength of skin specimens or healing wound scars [15,16], measurement of skin erythema by optical means [17-19], evaluation of skin water content by measurement of its dielectric constant [20] and examination of skin thickness by ultrasonic imaging [15,21]. Among the many methods, high resolution ultrasound of the skin has proven to be a precise and validated method used in many skin assessment studies following radiotherapy [21,22]. It enables accurate and easily reproducible determination of the skin's subcutaneous thickness [19] and allows real-time examination of the skin with relatively lower cost compared with other procedures such as biopsy and MRI.

Applications of ultrasound reported in dermatology are generally based on the measurements of skin thickness [23]. Such measurements have been applied to assess various skin conditions particularly fibrosis. Gottllober et al [24] used the change of skin thickness as an indicator of cutaneous fibrosis in their studies on five patients with cutaneous radiation syndrome. Huang et al [25] also reported a significant change of skin thickness in the head and neck region after radiotherapy using 20 MHz ultrasound. All these studies demonstrated the potential use of the ultrasound detection of skin thickness in assessing the postirradiation reactions of the skins. It is potentially helpful to use the ultrasonic properties to characterize the irradiated skin fibrosis because some changes of the skin structures are induced by therapeutic irradiation.

However, few clinical data were available in the literature for documenting the ultrasonic properties of fibrotic skin in vivo for postmastectomy breast cancer patients with radiation induced fibrosis. Therefore, this study aimed to (1) measure chronic skin changes quantitatively using ultrasound, in patients who have gone through postmastectomy irradiation, and (2) determine if there is any correlation between late skin findings and acute visible changes using the RTOG scoring criteria in postmastectomy patients.

## Materials and methods

### Patients

This study utilized the records of National Healthcare Group (NHG) from National University Hospital (NUH) and Tan Tock Seng Hospital (TTSH), Singapore to identify a group of female patients previously treated at these institutions for PMRT from January 2004-December 2005. Two hundred and five patient records were identified from the database. Of these, 7 patients had deceased, the data of 94 patients were not complete (absence of RTOG scoring in the five weeks of treatment), 26 were not contactable due to invalid addresses or telephone numbers, 16 patients had bilateral mastectomy, lumpectomy on the contra-lateral breast or metastases and 30 patients declined to participate in the study. This left 32 patients eligible to be invited to participate in the study. All of those patients were of Asian origin.

The primary criteria used to select patients from the data obtained from NUH and TTSH included a total mastectomy with no bilateral involvement and should have completed full course of radiotherapy and chemotherapy treatments. Radiotherapy dosages and RTOG scoring were explicitly specified on the fifth treatment of each of the five treatment weeks and six cycles of adjuvant intravenous chemotherapy; CMF (cyclophosphamide, methotrexate and 5-fluorouracil) were delivered following the radiotherapy. The contra-lateral breast which was not irradiated was categorized as control. Patients who had lumpectomy on the nonirradiated breast and breast reconstruction on one side or bilaterally, and who had previous radiotherapy to the chest wall, either as definitive treatment or as entry/exit dose from previous intra-thoracic malignancy radiotherapy were excluded from the study.

This investigation was approved by the institutional ethics review board -National Health Group (NHG) Domain-Specific Review Board (DSRB). Patient Information Sheet including the reasons and the details of the study as well as an invitation to participate in the study was mailed to the subjects. Patient's Informed Consent form was signed by the patient on the same day of the ultrasound scan, prior to the scan.

#### Radiation Therapy

**Figure 1:**
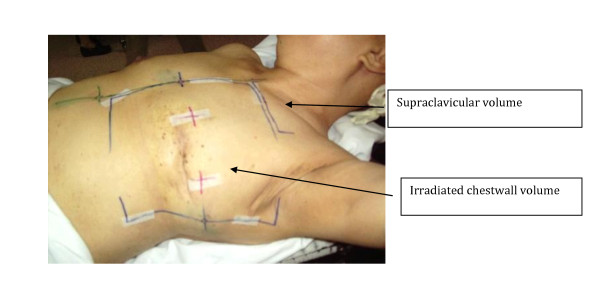
**Treatment marks on the patient**.

All patients were 3D planned using 3D Xio Treatment planning system. The patients were positioned supine with elevated arms, flexed elbows supported by a wingboard. A high pillow was used to support the knees to ensure patient fixation during radiotherapy. Prior to radiotherapy, all patients had treatment marks drawn using a permanent marker which extended from the second costal cartilage down to 1 cm inferior to the contra-lateral non-irradiated breast. The medial margin forms the midline and the lateral margin the mid-axillary line (Figure 1). Three permanent tattoos were used in the central treatment plane as a guide to reproduce the treatment marks, when necessary.

The whole breast had been irradiated using opposed tangential fields with 6 MV photons covering the axillary and the infra-and supra-clavicular areas for all patients. The total dose was 46-50 Gy administered in daily doses of 2 Gy 5 days a week using a Siemens Primus, linear accelerator (Siemens Medical Systems, USA). Wedges and bolus of 1 cm thickness were used every day in all patients to optimize the homogeneity of the dose distribution.

#### Ultrasound Measurements

To measure the skin thickness, ultrasound was performed using a Sequoia^® ^512 scanner (Siemens Medical Systems, USA) with a linear array transducer (15L8 W). The 52 mm foot print transducer has a wide bandwidth with a 14 MHz centre frequency and can achieve a maximum depth of 80 mm. The "Breast Detail" preset was selected to give the settings discussed in Table 1. All the scans were performed using a special magnification mode without substantial loss of resolution (RES-mode) to have clearer details and precise measurements of the skin.

**Table 1:** Relevant Equipment Settings for the 'Breast Detail' preset

Power	0dB, Mechanical index = 1.6: As Low As Reasonably Achievable to minimize bio-effects.
**Time Gain Compensation (TGC)**	Adjusted to compensate the effect of attenuation in the subcutaneous tissue at greater depths in order to produce images of uniform brightness.

**Dynamic Range**	66 dB permits best differentiation between subtle changes in echo intensities of the skin region.

**Persistence**	2 used to provide optimum smoothening of images with minimum movement induced artifacts.

**Post-processing**	Options range from low (0) to high (3) contrast. It was at 2 to obtain optimal level of contrast.

**Overall Gain**	Set at 2dB to demonstrate tissues with appropriate brightness.

**Edge**	+2 to emphasize the boundaries between tissues.

**Delta**	Δ2 for high contrast resolution.

**Spatial Compounding**	A form of frame averaging. SC1 selected to maximize frame rate.

**Frame Rate**	Decreases with increasing the scan depth, the number of focal zones and image line density (resolution). Varies during the scan but it should not be below 10 Hz (fps).

**Focal Zone**	Single focal zone placed at the level of the skin to optimize lateral resolution.

**Figure 2:**
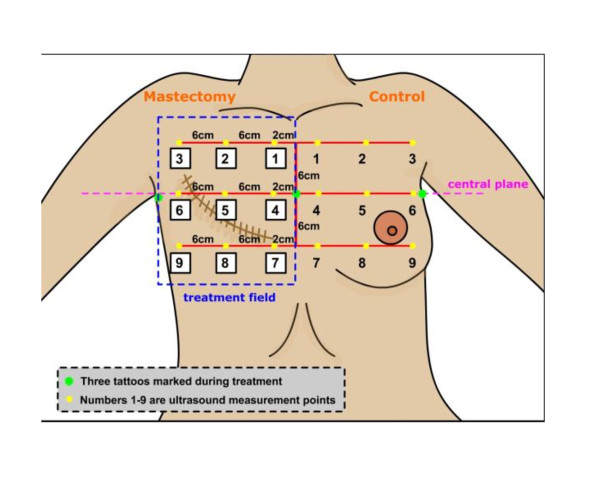
**Representation of points on the chest wall and contra-lateral non- irradiated breast**.

**Figure 3:**
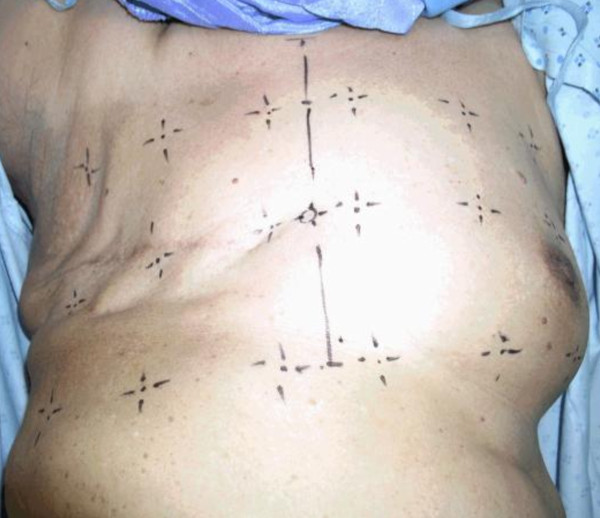
**Points marked on the chest wall and contra-lateral non-irradiated breast prior to ultrasound**.

After informed consent was taken, the patients were requested to lie supine on the couch. The treatment field points were reproduced with the aid of the patients' case notes and the presence of permanent tattoos which had been marked in the central treatment plane during the treatment. Nine points for ultrasonic measurements within the medial, central and the lateral areas of the treatment field as well as the corresponding points in the contra-lateral non-irradiated breast were marked as shown in Figure 2 and Figure 3.

**Figure 4:**
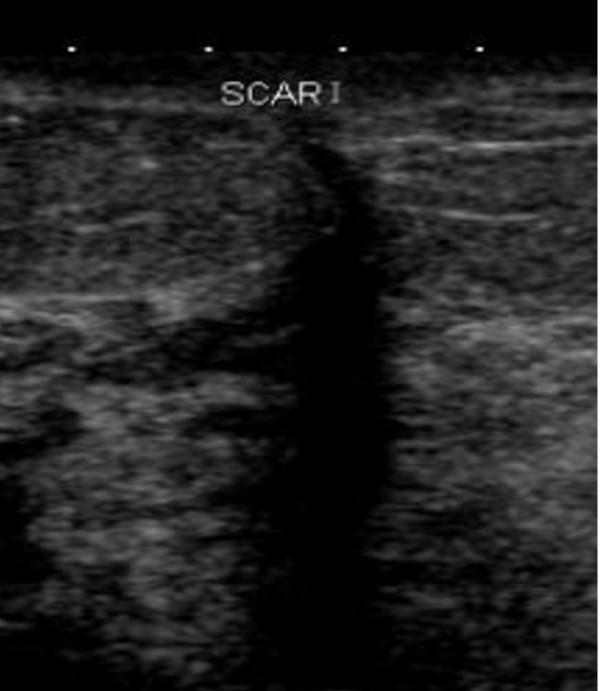
**Scar causes shadowing**.

A shadow found in the scar region can limit the ultrasound evaluation (Figure 4). If the measurement points fell on the mastectomy scar, the points were marked 1cm superiorly or inferiorly to the original points. The corresponding points were also marked on the contra-lateral non-irradiated breast to achieve measurement consistency.

**Figure 5:**
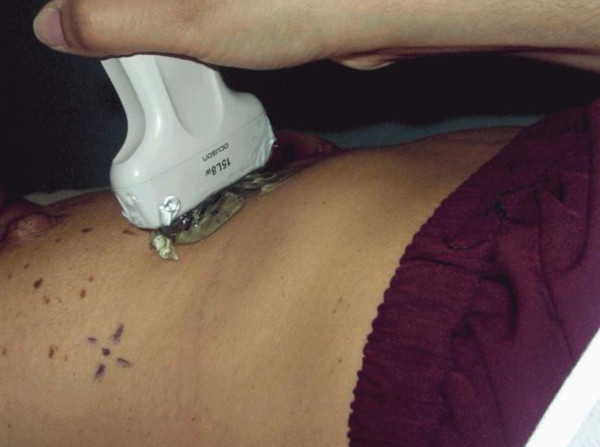
**Transducer resting on the thick layer of gel on the skin**.

All ultrasonic measurements were obtained using the slightest transducer force on the skin, by ensuring the transducer rested on the thick layer of gel to avoid affecting skin thickness (Figure 5). All patients were scanned by the same sonographer to reduce inter-operator error.

**Figure 6:**
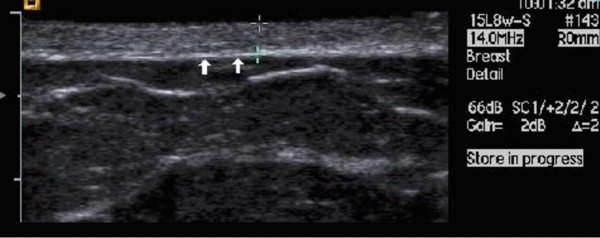
**Echogenic border between the skin and the subcutaneous tissue**.

The axis of the transducer was kept perpendicular to the surface of the skin while scanning to maximize specular reflection at the skin/subcutaneous tissue interface (Figure 6). All the points marked were individually scanned in the transverse plane. The full skin thickness (epidermis plus dermis) from the anterior echogenic border of the epidermis to the posterior echogenic border of the dermis was measured in B-mode. All images were stored in the hard-drive of the ultrasound machine for analyses. As a reference measurement, examination of the contra-lateral non-irradiated breast was carried out at the same time. This was a blind study whereby the sonographer was not aware of the retrospective collected data of the RTOG acute scoring.

### Statistical Analyses

The measurements of each of the nine points of irradiated skin were compared with corresponding points of non-irradiated skin by using a t-test. This test determined the significance of differences between the values of the same measurements made under the two different conditions (postmastectomy with radiation skin thickness versus non-irradiated contra-lateral skin thickness). These measurements obtained in the radiated breast were compared to the retrospective data of the peak acute RTOG scoring obtained during the treatment weeks by utilizing a t-test. A p value < 0.05 was considered significant.

## Results

The median age of patients at the time of ultrasound scan was 52.5 years (range 37 to 68 years). The median interval between the last radiotherapy treatment and current ultrasound scan was 27.5 months (range 16 to 39 months). The skin thickness was reduced in the irradiated chest wall compared to the contra-lateral non-irradiated breast (Table 2 and Table 3). The mean total skin thickness inclusive of the epidermis and the dermis of the right irradiated side was 0.1712 mm (± 0.03392 mm) compared with the left non-irradiated breast which was 0.1845 mm (± 0.04089 mm; p = 0.007). The left irradiated skin had a mean skin thickness of 0.1764 mm (± 0.03184 mm) compared with the right non-irradiated breast which was 0.1835 mm (± 0.02584 mm; p = 0.025). These independent t-tests produced a significant difference of reduced skin thickness on the right irradiated chest wall, p = 0.007 (p < 0.05) and left irradiated chest wall ρ = 0.025 (p < 0.025) in comparison to the non-irradiated skin thickness investigating chronic skin reactions.

**Table 2:** Reduced mean skin thickness on the Right mastectomy side with radiation in comparison to the Left non-irradiated breast.

STATISTICS					
	**Side**	**N**	**Mean**	**Std. Deviation**	**Std. Error Mean**

Skin thickness mm	Right Mastectomy	117	.1712	.03392	.00314
	Left Control	117	.1845	.04089	.00378

*The sample size N = 117 refers to the 9 measurement points taken in 13 Right Sided mastectomy patients.

**Table 3:** Reduced mean skin thickness on the Left mastectomy side with radiation in comparison to the Right non-irradiated breast

STATISTICS					
	**Side**	**N**	**Mean**	**Std. Deviation**	**Std. Error Mean**

Skin thickness mm	Left Mastectomy	171	.1764	.03184	.00243
	Right Control	171	.1835	.02584	.00198

* The sample size N = 171 refers to the 9 measurement points taken in 19 Left Sided mastectomy patients.

Skin thickness for grade 1 and grade 2 of skin toxicity was compared in this study. Other grades were not included as there was only one patient representing grade 0 and grade 3 respectively and none of grade 4. Patients with grade 2 acute skin toxicity presented with thinner skin compared to patients with grade 1. The mean skin thickness for patients who had grade 2 was 0.1720 mm (± 0.03132 mm), while for grade 1 it was 0.1879 mm (± 0.03900 mm, p = 0.006).

Measurements between irradiated and non-irradiated breast (contra-lateral), show that the skin on the medial aspect measured at points 1, 4 and 7 was consistently thicker than the lateral aspect measured at the marked points of 3, 6 and 9 (Table 4). A total of 3 points from both medial (points 1, 4 and 7) and lateral (points 3, 6, and 9) aspect of the breast was measured. Total N = 32 patients × 3 points (total) = 96 from each side.

**Table 4:** Skin thickness of points marked on the medial and lateral side

STATISTICS					
	**Side**	**N**	**Mean**	**Std. Deviation**	**Std. Error Mean**

Skin thickness on contralateral non-irradiated breast mm	Medial	96	.1947	.02440	.00249
	Lateral	96	.1773	.02988	.00305

Skin thickness on mastectomy irradiated side	Medial	96	.1960	.03330	.00340
	Lateral	96	.1669	.03716	.00379

*The sample size N = 96 refers to 3 measurement points taken from each side of the 32 patients

## Discussion

In this study we demonstrated quantitative ultrasound as an objective means of assessing late skin toxicity in postmastectomy breast cancer patients after radiation therapy. Huang et al [25] has demonstrated that skin thickness measured via ultrasonic imaging proved to be a reliable quantitative and noninvasive measure to provide diagnostically useful information for an in vivo assessment of skin fibrosis. Using this as a feasible study, skin thickness was measured as a proxy for radiation induced fibrosis and edema in 32 postmastectomy breast cancer patients who received full course of radiation therapy in our department. This is one of the few studies of its kind on postmastectomy breast cancer patients follow up comparing late skin effects particularly radiation induced fibrosis with acute scoring. To the best of our knowledge, there is limited literature available about radiation induced fibrosis after postmastectomy radiotherapy in ultrasonic imaging. This study attempted to provide some basic clinical results for this purpose. Our study has shown that there is a statistically significant difference between the skin thicknesses of the irradiated chest wall and the contra-lateral non-irradiated breast and a predisposition to chronic reactions was found in patients with acute RTOG scoring of grade 1 and grade 2. These differences could assist to explain breast reconstruction complications in postmastectomy breast cancer patients after receiving a course of radiotherapy. A number of studies have demonstrated an increased rate of breast reconstruction complications after postmastectomy radiotherapy [26-29]. Tallet et al [29] reported breast reconstruction complications were significantly greater in patients who received radiotherapy than those who did not (51% vs 14%) and a study by Behranwala et al [27] suggested that the rate of capsular contracture due to radiation might be as high as 40%. As such most complications were either due to capsular contracture with or without pain, or skin fibrosis due to radiation exposure.

In an effort to explain the increased breast reconstruction complications in patients due to increased radiation dose to the skin, a separate pilot study (results submitted for publication) was conducted in our department to assess skin dose in post-mastectomy radiation measured by TLDs in a customized chestwall phantom. The measurements were also analyzed with the use of wedges and the presence of bolus. The surface dose with bolus was determined to be much larger than that without bolus as expected (39% increase in dose when bolus was used).

In addition, results showed that the oblique incident angle and flat chestwall thickness also played an important factor in increasing the skin dose in postmastectomy patients. As severity of skin toxicity is radiation-dose related, this suggests a possible relationship with breast reconstruction complications and an impairment of the skin thickness with radiation due to skin fibrosis.

The accuracy of the ultrasonic measurement of skin thickness has been established since the late 1970s [23]. By introducing this method in our study, we found that the breast skin thickness decreased significantly in the postmastectomy patients after radiotherapy. In our study, the skin thickness of the postmastectomy breast cancer patients was approximately 9.2% to 9.6% smaller than that of the control non-irradiated side. This is in contrast to previous publications [24,30] that reported an increase of 37% in the neck region and 38% in the conservatively managed breast following irradiation. One possible reason for the discrepancies was the difference of radiation dosage used for the patients. Another possible reason was the difference of the follow-up time of the subjects at recruitment.

**Figure 7:**
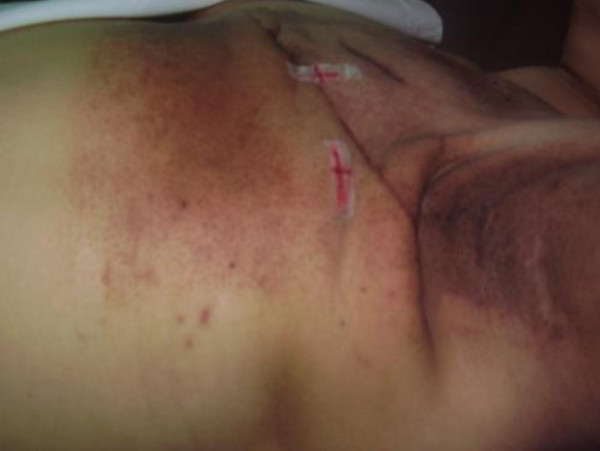
**Erythema and dry desquamation seen in Grade 1**.

Our proposed mechanism was supported by Warszawski et al [30] who reported that structural changes occurring after radiotherapy depend on the time interval between the completion of treatment and ultrasonic examination. Fibrosis is a common late side effect of radiotherapy treatment for cancer patients and is considered to be a dose-limiting factor. It has been reported that the latency of fibrosis is between 1-2 years post radiotherapy and the severity of fibrosis progresses over time. Usually the skin first becomes erythematous due to desquamation (Figure 7), then thinned due to inadequate cell proliferation in the basal layer [31]. If damage is too severe, the skin will break down and ulcerate due to depletion of the regenerating cells in the basal layer [32]. In a group of 106 patients with lumpectomy followed by radiotherapy were examined by Leucht et al using ultrasound, the time between completion of radiotherapy and the ultrasound examination ranged from 3 weeks to 8 years [33]. Skin thickness was noted to initially increase then decrease after 2 years. Another study reported by Calkins et al examined twenty-one breast cancer patients who had undergone segmental resection and radiation therapy [34]. The authors also observed an increase in skin thickness followed by a decrease in four of nine patients for whom serial ultrasound scans were performed beginning one to forty eight months after their radiation. A review of literature [34,35] have shown that majority of patients assessed were those following conservative surgery and irradiation.

### Correlations of Acute skin scoring (RTOG) and fibrotic skin thickness

Skin reactions occurring within a time interval of up to three months following irradiation are defined as early reactions and reactions presenting after three months following irradiation are defined as late reactions [7]. There is a well-established process of visual assessment of acute skin reactions using RTOG Scoring [35] which is carried out subjectively by the examining physician (Table 5). The acute toxicity analyzed in this study was the peak score recorded during the weekly assessment at the end of every radiotherapy treatment.

**Table 5:** RTOG Scoring Criteria for Acute Radiation Skin Reactions

RTOG Scoring Criteria	Skin Changes
**0**	No change over baseline

**1**	Follicular, faint or dull erythema, epilation, dry desquamation, decreased sweating

**2**	Tender or bright erythema, patchy moist desquamation, moderate oedema

**3**	Confluent, moist desquamation other than skin folds, pitting oedema

**4**	Ulceration haemorrhage, necrosis

In this study, grade 1 acute skin reaction was found in 72% of patients, grade 2 in 22% of patients and grade 3 and grade 0 were found in 3% each. No patient had grade 4 skin reaction in the study. These results are similar to those reported in literature whereby Back [36] demonstrated a low rate of acute toxicity with a RTOG score of 3 and 4 which occurred in only 6% of women receiving radiotherapy. Small and Woloschak [31] also added that on average, more than 80% of acute skin toxicity during breast radiation was in the grades of 1 and 2.

In our study we correlated acute RTOG with their measured skin thickness and demonstrated that patients with higher grade of acute skin reactions; i.e., grade 2, demonstrated reduced skin thickness as compared to those patients with grade 1 reaction in table 6.

**Table 6:** Reduced mean skin thickness in patients with grade 2 acute skin reactions

Group Statistics					
	**grade**	**N**	**Mean**	**Std. Deviation**	**Std. Error Mean**

Skin thickness	grade 1	207	.1879	.03900	.00271
mm	grade 2	63	.1720	.03132	.00426

*The sample size N = 207 refers to the 9 measurement points taken in 23 patients with Grade 1 and N = 63 refers to the 9 measurement points taken in 7 patients with Grade 2.

On ultrasound, the skin appears echogenic and is well demarcated from the underlying hypoechoic subcutaneous fat. However, the epidermis cannot be resolved from the dermis on ultrasound. The epidermis is composed of several layers. The stratum basale is the deepest layer where the majority of cell division occurs. This layer is the most sensitive for radiation injury that results in the clinically visible acute radiation skin reactions [37].

In addition, we also looked at the variation in skin thickness across the breast. The medial and lateral aspects of both sides were examined symmetrically. Our results showed that the skin on the medial aspect was consistently thicker than the lateral aspect on both the irradiated postmastectomy and non-irradiated side. This reported mean thickness is similar to previous studies by Wratten et al [38] that compared variation in skin thickness in the conservatively managed breast. This finding suggests a need to use the same examination point when performing serial and comparative examinations.

The results of this study may be limited because of small study population and non-representation of patients with all the grades of RTOG scoring. Our study has a low incidence of other grades of reactions especially higher grades and thus, it was not possible to investigate whether the higher grades (grade 3 and grade 4) showed a convincing trend of an increased probability of thinning in the skin thickness.

It is also possible that other factors such as the age, chemotherapy and surgery of the patients may have influenced the results. However, Turesson et al [17] studied 402 breast cancer patients who received radiotherapy and were followed up for ten years. Prognostic factors for acute and late skin reactions such as treatment-related variables and patient-related variables were analyzed. They could not verify that age; hemoglobin level, smoking and collagen vascular diseases had significant influence on the acute and late skin reactions.

High frequency ultrasound provides easy, low cost and quantitative value in studying skin thickness in acute and chronic skin reactions. A great attention to the ultrasound technique must be given. It is important to maintain the ultrasound beam perpendicular to the surface of the skin to avoid artifacts due to scattering and minimizing variations in pressure of the transducer on the skin which may alter its apparent skin thickness. In this study, each marked point was only measured once and may have affected the intra-rater variability. An average measurement of two to three times would be more representative of each of the measured point. However, Agner et al [39] reported that the coefficient of variation of repeated ultrasound measurements were low (approximately 2.2% on normal skin).

Further prospective studies are required in measuring ultrasonic skin thickness before, during and after radiotherapy treatment followed-up yearly after treatment for about 10 years to document quantitative skin changes. Warszawski et al [21] proved that the structural changes in the assessment of early and late skin reactions can be recorded by ultrasonic evaluation and much earlier than visible reactions by the naked eye of the examining physicians. However, results did not demonstrate any significant difference in skin changes over long periods.

Patients requiring radiotherapy had a higher rate of expander implant breast reconstruction failure and complications to patients who did not receive RT [40]. This can be explained in part by increased thinning of the skin post radiotherapy which increased overall contracture failure rate and thus, adverse cosmetic outcome.

Further studies are needed to confirm this explanation by quantifying the changes of the water and collagen in post irradiated skin. Two directions may be considered in future investigations to study how the severity of fibrosis affects skin thickness. One is to conduct biochemical or histological examinations directly to quantify the level of skin fibrosis and the other is to measure specifically the physical properties such as elasticity of the skin layer, as an indicator of the cutaneous fibrosis before and after radiation. This leads us to our next study which looks at histological changes of human skin cells after fractionated radiotherapy in an effort to explain the effects of fibrosis and skin changes in postmastectomy radiotherapy.

## Conclusion

Our study has proven that high frequency ultrasound can be utilized to document quantitative sonographic changes of the skin thickness in postmastectomy breast cancer patients following radiotherapy. This study also demonstrated a statistically significant difference in the skin thickness between the irradiated side and the contra-lateral non-irradiated breast. This finding might be helpful to predict late skin reactions. In spite of just using two grades of RTOG scoring in this study, a statistically significant predisposition to chronic reactions was found in patients with acute radiation reaction. The ability to successfully predict the complication risk for individual patients can lead to real advances in radiation oncology. These results can be used to decide which patients would benefit from breast reconstruction and perhaps dissuade patients whose skin is too thin and who are likely to experience breast reconstruction failure after postmastectomy radiotherapy.

## Competing interests

The authors declare that they have no competing interests.

## Authors' contributions

SW designed and participated in the development of the study, collected the data, performed the literature research and wrote the manuscript. AK performed the sonography examinations and performed the statistical analysis. MB helped in the designing of the study, participated in literature research and revision of the manuscript. JL was the study supervisor who gave guidance on the study and participated in the writing and revision of the manuscript. All authors have read and approved the final manuscript.

## Patient'S Consent

Written informed consent was obtained from the patient for publication of this case report and accompanying images.
