# An ultrasonographic evaluation of skin thickness in breast cancer patients after postmastectomy radiation therapy

**DOI:** 10.1186/1748-717X-6-18

**Published:** 2011-02-18

**Authors:** Sharon Wong, Amarjit Kaur, Michael Back, Khai Mun Lee, Shaun Baggarley, Jiade Jay Lu

**Affiliations:** 1 National University of Singapore, Yong Loo Lin School of Medicine, 21 Lower Kent Ridge Road, Singapore 119077; 2 Nanyang Polytechnic, School of Health Sciences, 180 Ang Mo Kio Avenue 8 Singapore 569830; 3 Northern Sydney Cancer Centre, Royal North Shore Hospital, St Leonards, New South Wales 2065, Australia; 4 National University Cancer Institute, Department of Radiation Oncology, National University of Singapore, 1E Kent Ridge Road, Tower Block, Level 7, Singapore 119 228

## Correction

Following publication of our article [1], we noticed that the skin thickness measurement units stated in the manuscript were incorrect. The correct units of measurement is centimeters (cm) and not millimeters (mm) as indicated in the article. This, however, does not change the statistical findings.
